# Exploring treatment specifics of addictive disorder in a young adult living in a post-war middle income country with rapid social and cultural transition: a qualitative case report

**DOI:** 10.1192/j.eurpsy.2024.840

**Published:** 2024-08-27

**Authors:** N. Agani

**Affiliations:** of Psychiatry, University Clinical Centre of Kosova, Pristina, Kosovo

## Abstract

**Introduction:**

Addictive disorder, characterized by the tendency to abuse an illicit substance or manifest a repeated risky behavior, is a fairly common phenomenon occurring in the last 50 years, predominantly in middle and high income countries. While psychotherapy has an evident positive impact in the treatment of the disorder, data has shown that it is often not sufficient to achieve full remission and have optimal positive impact in the quality of life compared to simultaneous use of psychotherapy, pharmacotherapy and psychosocial rehabilitation.

**Objectives:**

The aim of the study is to highlight different specifics of the inpatient treatment of “Mr. E”, living in a post-war middle-income country with rapid social and cultural transition.

**Methods:**

Subject of this case study is “Mr. E” a 17 year old student with a history of family trauma with a long history of abuse and ambulatory psychiatric treatment. Data has been analyzed from the medical history of the patient treated in 2023, in the substance abuse unit of the Department of Psychiatry, University Clinical Center of Kosova. Semi structured interviews, daily abstinence symptoms evaluation and self - report measures were used to gather qualitative data throughout the treatment process. Treatment protocol consisted on: detoxification, pharmacotherapy and simultaneous supportive individual and group psychotherapy, with the goal to evaluate, treat and reintegrate “Mr. E” into the society free of illicit substance abuse. Comorbidities are correlated with underlying causes, while a healthy lifestyles are promoted through the work on behavior changes that will support optimal social reintegration in a rapid changing social and cultural environment.

**Results:**

The findings revealed several significant therapeutic objectives such as: Enhanced self – awareness; Reduced ruminations and increased self-control; Enhanced quality of life; and Decreased substance abuse. Detoxification protocol in the treatment of addictive disorder in inpatient psychiatric treatment was essential for abstinence symptom management during crisis. Strengthening the body parallel to healing the mind was found as an important stepping stone.

**Conclusions:**

Combined, detoxification, psychopharmacological, and psychotherapeutic approach was essential for successful treatment of a young adult in a post-war middle income country with rapid social and cultural transition.

**Disclosure of Interest:**

None Declared

